# Detecting ligand interactions in real time on living bacterial cells

**DOI:** 10.1007/s00253-018-8919-3

**Published:** 2018-03-17

**Authors:** João Crispim Encarnação, Tim Schulte, Adnane Achour, Hanna Björkelund, Karl Andersson

**Affiliations:** 1 Ridgeview Instruments AB, Vänge, Sweden; 2 Department of Immunology, Pathology and Genetics, Uppsala University, Uppsala, Sweden; 3 Science for Life Laboratory, Department of Medicine Solna, Karolinska Institute, and Department of Infectious Diseases, Karolinska University Hospital, Solna, Sweden

**Keywords:** Real-time interactions, Drug kinetics, Living bacteria, Antibodies

## Abstract

Time-resolved analysis assays of receptor-ligand interactions are fundamental in basic research and drug discovery. Adequate methods are well developed for the analysis of recombinant proteins such as antibody-antigen interactions. However, assays for time-resolved ligand-binding processes on living cells are still rare, in particular within microbiology. In this report, the real-time cell-binding assay (RT-CBA) technology LigandTracer®, originally designed for mammalian cell culture, was extended to cover Gram-positive and Gram-negative bacteria. This required the development of new immobilization methods for bacteria, since LigandTracer depends on cells being firmly attached to a Petri dish. The evaluated *Escherichia coli* CJ236 and BL21 as well as *Staphylococcus carnosus* TM300 strains were immobilized to plastic Petri dishes using antibody capture, allowing us to depict kinetic binding traces of fluorescently labeled antibodies directed against surface-displayed bacterial proteins for as long as 10–15 h. Interaction parameters, such as the affinity and kinetic constants, could be estimated with high precision (coefficient of variation 9–44%) and the bacteria stayed viable for at least 16 h. The other tested attachment protocols were inferior to the antibody capture approach. Our attachment protocol is generic and could potentially also be applied to other assays and purposes.

**Electronic supplementary material:**

The online version of this article (10.1007/s00253-018-8919-3) contains supplementary material, which is available to authorized users.

## Introduction

Today we are facing a new era of antibiotic resistance, in which antibiotics are losing efficiency against previously controlled bacterial strains. New strategies are needed at the molecular level to characterize multidrug-resistant (MDR) strains in order to identify novel antimicrobial drugs (Nikaido 2009). Exploring the immune system against these MDR strains is a promising approach, including the development of antibodies against bacterial growth molecules or cellular efflux pumps. Antibodies against bacterial antigens may also be applied as diagnostic tools, to potentially help the clinics to faster and more accurately choose the proper treatment (Saylor et al. 2010; Boyles and Wasserman 2015). For this purpose, technologies that allow the characterization of molecular interactions and that can decrease the costs of these strategies would be highly beneficial for society.

Binding assays are fundamental for the characterization of receptor-ligand interactions and to understand their underlying molecular mechanisms (Hulme and Trevethick 2010; Keskin et al. 2016). There are many methods to choose from if the sole purpose is to obtain the affinity value of an interaction, which describes at which concentration half of the receptors are occupied at equilibrium. However, additional interaction characteristics may be highly relevant for a drug. For example, information about the kinetics such as the association and dissociation rates provides insight into suitable dose levels, administration, and residence time. Therefore, time-resolved assays such as surface plasmon resonance (SPR) have provided an important contribution for understanding interactions between ligands and proteins and for screening potential new drugs (Renaud et al. 2016). While such biophysical assays provide essential information and parameters for the interaction of isolated molecules in a simplified system, the same molecules might act differently in more complex intra- or extracellular environments with altered binding parameters (Andersson 2013; Renaud et al. 2016).

With the increase of different and advanced microscopy techniques, new assays, such as fluorescence cross-correlation spectroscopy, have emerged that expand our comprehension about interactions in cells (Bacia et al. 2006; Ma et al. 2014). Moreover, methods that are based on the principles of Förster resonance energy transfer (FRET), bimolecular fluorescence complementation, split-luciferase complementation, and the yeast two-hybrid system have enabled the detection of intra- and extracellular protein-protein interactions (Xing et al. 2016). However, some of these approaches are misleading with a relatively high ratio of false-positives, especially when high-throughput sampling is involved (Piehler 2005).

LigandTracer is a series of instruments primarily designed to study the interaction between proteins and living cells, in real time. This real-time cell-binding assay (RT-CBA) technology relies on the continuous detection of a fluorescently or radioactively labeled ligand in a target area and in a reference area on a regular Petri dish, which is mounted on a rotating support. This results in a reference-subtracted real-time binding curve that provides information about the affinity, kinetics, and possibly also the binding mechanisms of the interaction between the ligand and the cells within the target area. The LigandTracer instrument series have previously been used in an increasing number of studies (> 70 papers as of August 2017), primarily with protein ligands and living mammalian cells expressing target receptors, but alternative ligands and targets are possible, such as small molecular drugs, nanoparticles, and antigens on magnetic beads (Björkelund et al. 2011; Pa̧zik et al. 2011; Mihaylova et al. 2014; Li et al. 2017; Zeilinger et al. 2017; Bugaytsova et al. 2017). A major requirement for the use of the LigandTracer instrument is the necessity to immobilize cells or the isolated receptor on the surface of a regular Petri dish. While the immobilization of adherent eukaryotic cell lines or the passive adsorption of proteins to plastic surfaces is straightforward and well established, the immobilization of Gram-positive or Gram-negative bacteria is a non-trivial process. For instance, for microarrays that use bacterial cells as biosensors, it is crucial that cells are stably attached at the surface. However, the detachment of bacterial cells on these microarrays is still one of the major challenges, which results in unreliable data and sensor failure. One of the crucial factors for this defective attachment of bacterial cells is the very small contact area with the substrate surface relative to eukaryotic cells. Different approaches for bacterial immobilization have been presented, such as physical entrapment or non-specific binding of bacterial cells on chemically modified surfaces (Suo et al. 2010). However, these entrapment methods using substrates modified with chemicals, such as polylysine, gelatin, and alginate, suffer from weak binding and posterior detachment from the surfaces. Other studies have presented the use of antibody-modified gold or silicon chips as sensors to detect *Salmonella Typhimurium* and *Pseudomonas aeruginosa*, demonstrating the possibility of implementing bacteria-binding antibodies for improving attachment (Jenkins et al. 2004; Oh et al. 2004).

In this study, we applied antibody-based immobilization of bacteria to expand the application of LigandTracer to measure the interaction of ligands to receptors on bacterial surfaces. This approach was significantly more effective and reliable compared to all the other previously tested methods such as cell culture dishes and fibronectin- or poly-D-lysine (PDL)-coated surfaces. Although the end goal in the present work was to be able to follow ligand/bacteria interactions in real time with LigandTracer, the obtained immobilization methods may be implemented in other microbiological assays as well.

## Material and methods

### Bacteria

Two Gram-negative strains of *Escherichia coli* were used, the K-12 strain CJ236 and the B strain BL21(DE3). The bacteria were inoculated from a frozen bacteria stock. For every experiment, the bacteria were inoculated into LB liquid medium without antibiotics and shaken at 150 rpm at 37 °C overnight before the measurement.

For the experiments with *Staphylococcus carnosus*, overnight cultures of TM300 strains were grown in M9 medium as previously described (Schulte et al. 2016). Cells were pelleted by centrifugation (7000*g*, 5 min), re-suspended in PBS and adjusted to an OD600 of about 2.

### Fluorescence labeling

#### Antibody labeling

The rabbit polyclonal antibody to the *E. coli* O and K antigen (ab31499, Abcam, Cambridge, UK, denoted Ab99) was labeled with fluorescein isothiocyanate (FITC, F3651, Sigma-Aldrich, St. Louis, MO, USA) at the primary amines, i.e., lysines. This was performed as previously described (Stenberg et al. 2014), but with a preceding buffer exchange to borate buffer at pH 9 in order to remove any NaN_3_ that may affect the labeling process. The labeled antibody was purified through a NAP-5 column (GE Healthcare, Little Chalfont, UK) in phosphate buffered saline (PBS) for the exclusion of unbound fluorophore. The FITC-labeled polyclonal anti-BR antibodies were produced as previously described (Schulte et al. 2016) and the monoclonal FITC-labeled anti-His antibody was purchased (MA1-81891, Thermo Fisher Scientific, Waltham, MA, USA).

#### Bacteria labeling

Bacteria were labeled with SYTO-9 or FITC to evaluate the attachment efficacy in the dish over time. As a first approach, 4 × 10^8^ cells were labeled with SYTO-9 dye from the Dead/Live Bacterial viability kit (L7012, Thermo Fisher Scientific) and cells were labeled according to the manufacturer’s instructions. Excess dye was removed by washing the cells three times by centrifugation at 4000*g* during 5 min.

Prior to labeling with FITC (F3651, Sigma-Aldrich), 4 × 10^8^ cells were centrifuged at 4000*g* during 5 min for removal of medium. After that, cells were incubated with FITC (1 μg/μl) in 500 μL of borate buffer pH 9, for 1 h at 37 °C. To remove excess FITC, the cells were washed three times by centrifugation at 4000*g* during 5 min.

### Attachment procedures

#### Dish coating

The rabbit anti-*E. coli* antibody Ab99 and fibronectin (F0895, Sigma-Aldrich), both in PBS, were adsorbed to polystyrene Petri dishes (Nunc™, 263991, Thermo Fisher Scientific) at concentrations of 6 and 30 μg/mL, respectively, for 3 h at room temperature (RT). Similarly, poly-D-lysine (P6407, Sigma-Aldrich, denoted PDL) was coated to polystyrene dishes at RT in Milli-Q water for 1 h, at a concentration of 0.1 mg/mL. For antibody-based immobilization of *S. carnosus* to the target areas on the Petri dish, monoclonal THEHis antibody (A00186, GeneScript) was spotted on the selected target area at a concentration of 2.5 μg/mL, and incubated overnight at 4 °C. All solutions were added as 500 μL drops in ~ 3 cm^2^ areas, typically three areas per dish. After incubation, the remaining solutions were removed and the dishes washed with PBS.

#### Attachment of bacteria

500 μL of *E. coli* suspension with an optical density at 600 nM (OD600) of 1.0 (which approximately corresponds to 8 × 10^8^ cells per milliliter) were incubated for 1 h at 37 °C to the coated spots (Ab99, fibronectin, PDL) or directly to polystyrene dishes or cell culture dishes (Nunclon™, Cat. No. 150350, Thermo Fisher Scientific). *S. carnosus* were suspended in PBS at OD600 of about 2 and incubated on the target area for 1 h at RT. Excess of non-attached cells (*E. coli* and *S. carnosus*) was removed and the dishes washed with PBS, followed by a 30 min blocking with 1% bovine serum albumin (Sigma-Aldrich) in PBS to reduce unspecific ligand binding during the subsequent measurements in LigandTracer.

### RT-CBA measurements

The interactions between FITC-labeled antibody and the− attached living bacteria cells were measured in a RT-CBA with LigandTracer® Green (Ridgeview Instruments AB, Vänge, Sweden), using a blue (488 nm)–green (535 nm) detector. The background signal of the fluorescent antibodies was corrected for through reference subtraction, using uncoated (*E. coli* measurements) or anti-His antibody-coated (*S. carnosus* measurements) bacteria-free areas of the Petri dishes as references. All LigandTracer measurements were conducted in PBS at RT and started with a short baseline measurement in the absence of a labeled antibody, to detect the background signal. The antibodies were added stepwise to obtain kinetic information at different concentrations which improves the accuracy of subsequent curve fitting (Onell and Andersson, 2005). In some of the measurements, the antibody solution was replaced with fresh PBS to assess the dissociation process.

A set of control experiments were performed to study the properties of Ab99. The anti-human epidermal growth factor receptor antibody cetuximab (purification from Erbitux, Apoteket AB, Solna, Sweden) was used as a negative control. Additionally, the binding of Alexa 488-labeled goat anti-rabbit IgG antibody (ab150077, Abcam) to adsorbed Ab99 was detected to study the adsorption properties.

### Confocal experiments

Bacterial cells were immobilized to non-treated μ-slides (80821, Ibidi, Martinsried, Germany) for immunofluorescence studies using the same attachment procedures as described above, or to tissue culture-treated slides (80826, Ibidi). Images were taken immediately after bacteria attachment or after an overnight incubation in PBS on a rocker, mimicking the conditions in LigandTracer.

For the viability assay, the bacteria cells were stained with Dead/Live Bacterial viability kit (L7012, ThermoFisher) according to the manufacturer’s protocols. Slides were captured with Zeiss LSM 700 confocal microscope. Images from live and dead cells were processed and counted using ImageJ software (U. S. National Institutes of Health, Bethesda, Maryland, USA).

The white and green channel images of *S. carnosus* were taken after the LigandTracer experiments using the ZOE Fluorescent Cell Imager (Biorad, USA).

### Data analysis

The real-time binding curves produced in LigandTracer Green were analyzed in the evaluation software TraceDrawer 1.8 (Ridgeview Instruments AB, Vänge, Sweden). Data were normalized to compare the curvature, and curve intervals were created to extract the signal height at given time points of the curves. Binding curves depicting the interactions between the antibodies and bacteria attached were fitted to the OneToOne interaction model, which describes one monovalent ligand binding to one monovalent target.

### Data simulation

Binding curves based on the estimated kinetics of the FITC-Ab99–*E. coli* interaction (*k*_a_ 3.2 × 10^3^ M^−1^ s^−1^, *k*_d_ 5.2 × 10^−6^ s^−1^) were simulated in MATLAB 2015A (MathWorks, Natick, MA, USA). The simulations assumed a linear decrease of the total amount to targets at varying degrees (0, 3, 10, 30, and 50%) over a 20-h detection period. The maximum signal *B*_max_, representing complete target saturation, was set to 100 as a starting value.

## Results

### Immobilization of Gram-negative *E. coli* to adsorbed antibodies for subsequent LigandTracer measurements of antibody-receptor interactions

**Fig. 1:**
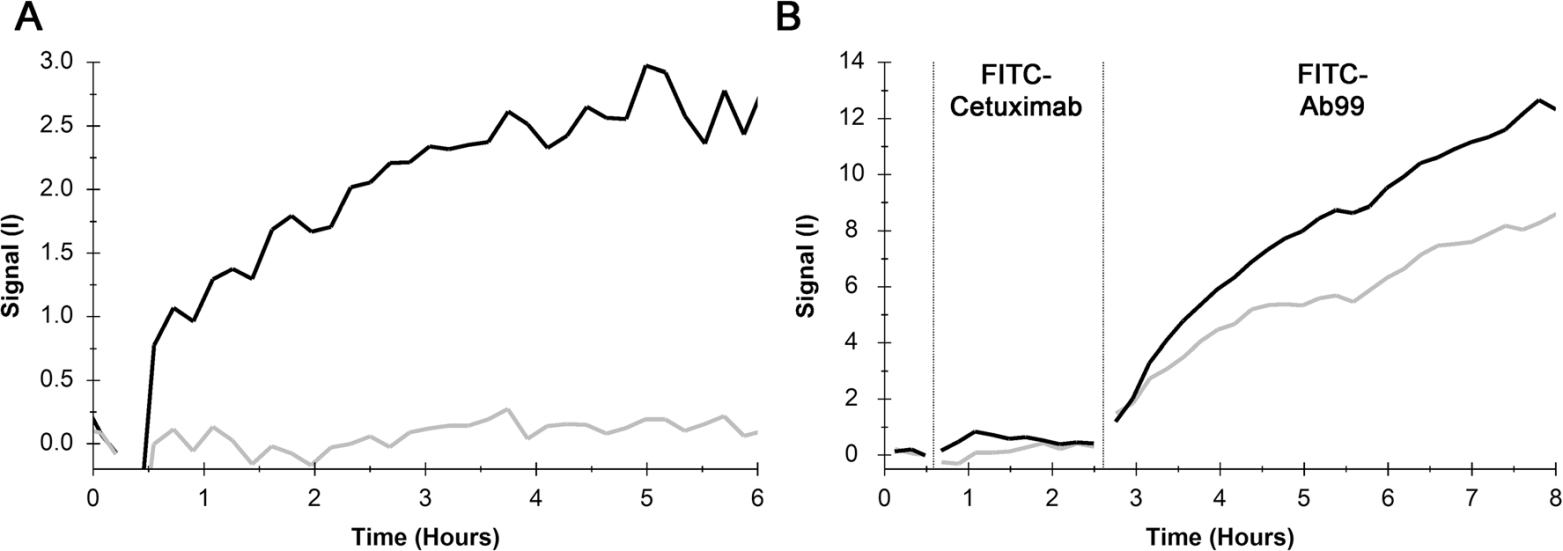
**a** FITC-Ab99 interacting with BL21 attached with adsorbed Ab99 (black) or to adsorbed Ab99 without bacteria, measured simultaneously. No binding to adsorbed FITC-Ab99 was observed. **b** FITC-cetuximab and FITC-Ab99 interacting with Ab99 attached *E. coli* of the strains CJ236 (black) and BL21 (gray), followed by the addition of FITC-Ab99 to confirm the presence of bacteria

After having established passive adsorption of an anti-*E. coli* antibody (ab31499, denoted Ab99) to the surface of a Petri dish (Supplemental Fig. S1A), the immobilization of the *E. coli* strain BL21 to the Ab99 spots was optimized with final incubation times of 3 h and cells at optical densities of 1.0 (Supplemental Fig. S1B). Although longer incubation times could theoretically increase binding levels, we observed a reduced biological activity of Ab99 after very long incubation times (> 1 day, data not shown) and therefore decided for 3-h adsorption protocols. The specific interaction of FITC-Ab99 with the immobilized bacteria was confirmed in two control experiments. In the first experiment, FITC-Ab99 only bound to the spot with immobilized bacteria (Fig. 1a, black) but not to the bacteria-free spot (Fig. 1a, gray). In the second control experiment, the FITC-labeled monoclonal antibody against the human epidermal growth factor receptor (cetuximab) did not bind to either attached CJ236 or BL21 bacteria (Fig. 1b). Subsequent incubation with FITC-Ab99 resulted in detectable binding signals, confirming the presence of immobilized bacteria during these experiments. The labeling and biological functionality of cetuximab was confirmed in a separate experiment (data not shown).

**Fig. 2:**
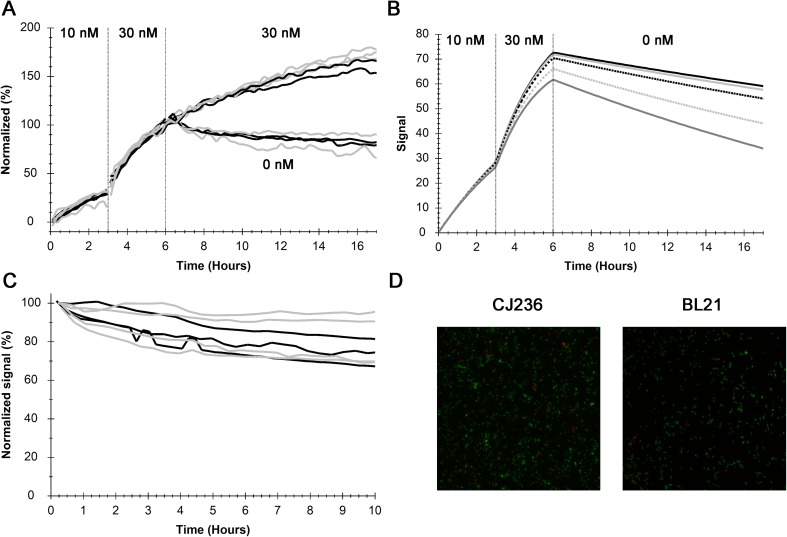
**a** Association and dissociation of FITC-labeled Ab99 to *E. coli* strains CJ236 (black) and BL21 (gray) attached with adsorbed antibody, in duplicates or triplicates for each combination of strain and incubation variant (with/without dissociation phase). The curves were normalized, setting 0% as the start of the 10 nM incubation and 100% as the end of the 30 nM incubation. **b** Simulations of how the binding curves would appear with a linear detachment of in total 0% (black, solid), 3% (light gray, solid), 10% (black, dotted), 30% (light gray, dotted), and 50% (dark gray, solid) of the bacteria over a 20 h period. Kinetic constants based on data from (A), assuming 0% detachment. **c** Signal decrease of FITC-labeled *E. coli* strains CJ236 (black) and BL21 (gray) when attached to adsorbed Ab99. After 10 h of measurement in LigandTracer 74% (± 6%, *n* = 3) or 81% (± 12%, *n* = 4) of the signal remained for CJ236 and for BL21 respectively. **d** Confocal images after 16 h on a rocker, when attached to adsorbed anti-*E. coli* antibody (Ab99). The bacteria were stained with a viability kit and the green and red colors represent the living and dead cells, respectively

After having established stable immobilization of the bacteria on the Petri dish surface, and the specific interaction of FITC-Ab99 with the surface-accessible bacterial antigen, the antibody-bacteria interaction was investigated more thoroughly using LigandTracer Green (Fig. 2a). Although lower signals (~ 50%) were obtained with BL21 compared to CJ236, likely due to some differences in antigen expression, the normalized binding curves revealed similar and repeatable interaction kinetics. The CJ236 binding curves were fitted using a OneToOne model, yielding kinetic constants of *k*_a_ 3.2 ± 0.7 × 10^3^ M^−1^ s^−1^ and *k*_d_ 5.2 ± 1.0 × 10^−6^ s^−1^ for the association and dissociation, respectively, with a derived affinity value of *K*_D_ 1.6 ± 0.7 nM. These values were then used to simulate the progression of the kinetic curves under the condition that a proportion of the bacteria would detach over time (Fig. 2b—assuming 0% detachment in the curves of Fig. 2a). If many bacteria would detach, the signal would decrease significantly over time (Fig. 2b), visible as more pronounced curvature during incubation and a faster off-rate in the dissociation phase. However, such a strong decrease was not observed in the measurements (Fig. 2a). Instead, the uptake of antibody was slow, with only little detectable curvature during the association phase. The signal decreased approximately 25% during the first 10 h of the dissociation measurement, due to dissociation of FITC-Ab99 from the bacteria as well as potential bacteria detachment from the dish.

To investigate the stability of the attachment, the *E. coli* bacteria were labeled fluorescently, attached to the dishes according to the defined protocols and then measured overnight. Any signal decrease should be directly related to bacteria detachment, in contrast to the indirect measurements of attachment described above in which the dissociation of FITC-Ab99 also reduced the signal. In a first test, the bacteria were labeled with SYTO-9 which produced high signals. However, the signal decreased as much as 90% over the first 5 h of measurement. Since this was much faster than what could be interpreted from the measurements with FITC-Ab99, the bacteria was instead labeled with FITC. Assuming that the fluorescent labeling of bacteria was stable, this would correspond to the amount of attached bacteria. The signals after 10 h were 74% (± 6%, *n* = 3) for CJ236 and 81% (± 12%, *n* = 4) for BL21.

Due to measurement times of several hours in LigandTracer experiments, bacteria were also checked for viability in confocal imaging assays that were performed under similar conditions as in a typical LigandTracer run. Confocal images were taken of *E. coli* immobilized on a polymer coverslip with Ab99, and incubated with a live-dead stain as viability indicator. These plastic microscope slides have optical properties similar to glass and allowed us to mimic the conditions of a polystyrene Petri dish. Images were taken immediately after bacteria attachment (0 h) and after 16 h incubation on a rocking shaker to represent an overnight LigandTracer run. The number of bacteria and the viability were almost identical at 0 h (data not shown) and 16 h incubation times (91.8 and 96.2% of CJ236 and BL21 live cells, respectively). The wells with BL21 contained fewer cells than the CJ236 wells, which was in line with previous signal differences of the FITC-Ab99 binding as detected by LigandTracer. The bacteria were evenly distributed and did not form any obvious clusters, but there were some signs of mitosis.

In summary, these results demonstrate that Gram-negative bacteria can be immobilized on the surface of Petri dishes through the use of passively adsorbed antibodies, allowing reproducible determination of the kinetics and affinity values of receptor-ligand interactions on Gram-negative bacteria as evaluated in RT-CBA.

### Antibody-based immobilization of Gram-positive *S. carnosus* for LigandTracer measurements of antibody-receptor interactions

**Fig. 3:**
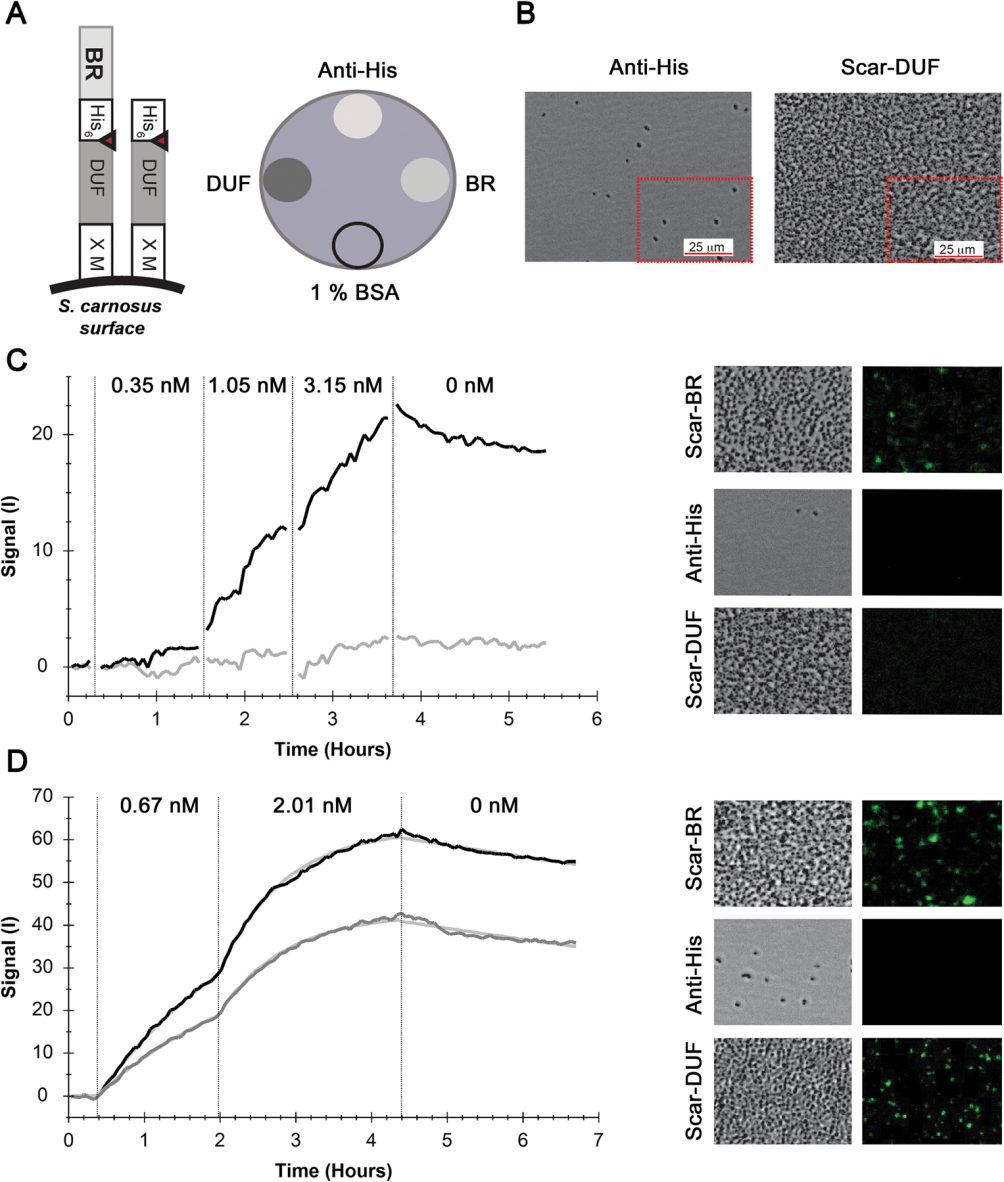
Affinity determination of the interaction between FITC-labeled antibody and surface-displayed antigens of *S. carnosus* in LigandTracer experiments. **a** The two S*. carnusus* strains display either the DUF linker domain alone (Scar-DUF) or in fusion with the BR domain of PsrP (Scar-BR) on the bacterial surface. Proteins are covalently coupled to the peptidoglycan through a Sortase recognition motif (X M). The His-tag for surface immobilization and affinity determination is the N-terminal of the DUF-linker, or the C-terminal of the BR domain. The anti-His antibody for immobilization was spotted on three target areas, of which two were loaded separately with Scar-DUF (gray) or Scar-BR (light gray). **b** Phase-contrast microscopy images demonstrated that target areas were densely covered with bacteria (Scar-DUF) while few probably detached bacteria were captured on the anti-His surface after the LigandTracer experiment. **c** The polyclonal affinity-purified FITC-labeled anti-BR antibodies clearly bound to Scar-BR (black), but not, or minimally, to Scar-DUF (gray). The white and green channel images were taken after the binding experiment using a phase-like contrast microscope, demonstrating FITC-staining of the immobilized Scar-BR, but not Scar-DUF or the anti-His antibody control. **d** The monoclonal FITC-labeled anti-His antibody bound to Scar-BR (gray) and Scar-DUF (black). The curves were fitted using a one-to-one binding model (light gray), which resulted in *K*_D_ values of 158 ± 14 pM (*n* = 2) and 120 ± 16 nM. (*n* = 2) for Scar-BR and Scar-DUF respectively. The white and green channel images were taken using a phase-like contrast microscope after the experiment, demonstrating FITC-staining of the immobilized Scar-DUF and Scar-BR bacteria, but not the antibody control

The same antibody-based immobilization strategy was then applied to the Gram-positive bacterium *S. carnosus*, representing a well-established model system to display protein receptors on the bacterial surface (Samuelson et al. 2002; Kronqvist et al. 2008). The available surface-display vector of *S. carnosus* is constructed such that the gene of interest is fused to a domain of unknown function (DUF) linker domain and becomes covalently attached to the staphylococcal cell wall through Sortase-mediated enzymatic linkage (Fig. 3a). Furthermore, a fused hexa-histidine-tag between the DUF linker and the gene of interest allows the use of anti-His-directed antibodies. The binding region (BR)_187–385_ domain of the pneumococcal serine-rich repeat protein was chosen as surface receptor since we have recently characterized this domain using the described surface-display vector with specific anti-BR polyclonal antibodies (Schulte et al. 2016).

Two *S. carnosus* constructs that display either the DUF linker alone (Scar-DUF) or the DUF linker with the BR_187–385_ domain (Scar-BR_187–385_) were immobilized with adsorbed anti-His antibody at high density on the target surface, as revealed by phase-contrast microscopy images taken after the LigandTracer binding experiments (Fig. 3a, b). Only few cells detached and were bound on the anti-His control surface (Fig. 3b, c). However, the low number of detached cells caused minor, if any, cross-contamination of the surfaces since the FITC-labeled polyclonal antibodies raised against the BR_187–385_ domain only bound to the attached Scar-BR_187–385_, but only minimally to the attached Scar-DUF (Fig. 3c) or the anti-His control surface (data not shown). Since the polyclonal antibodies comprise a mixture of high- and low-affinity binders, the data were not fitted using any of the provided binding models.

Altogether, these results indicated that the setup was suitable to determine the affinity of the interaction between a fluorescently labeled ligand and a receptor displayed on the bacterial surface. Indeed, kinetic binding curves were obtained for binding of a monoclonal FITC-labeled anti-His antibody to both Scar-BR_187–385_ and Scar-DUF that were fit using a one-to-one binding model yielding *K*_D_ values of 158 ± 14 pM and 120 ± 16 pM, respectively. The difference was not statistically significant (unpaired Student’s *t* test, *p* = 0.05). Furthermore, the dissociation signal decreased only 25 and 28% from the Scar-BR_187–385_ and Scar-DUF areas, respectively, similarly to the previously described *E. coli* measurements. Therefore, we conclude that the antibody-based immobilization strategy is also suitable to analyze receptor-ligand interactions on the surface of Gram-positive bacteria.

### Alternative attachment methods

Aside from the utilization of adsorbed antibody, other immobilization methods such as the use of cell culture dishes as well as fibronectin and poly-D-lysine (PDL) coatings were tested in order to assess whether these methods could compete with the described adsorbed antibody approach. However, our tests indicated that both fibronectin and PDL coatings could not be used for real-time interaction LigandTracer measurements (Supplementary material). The main drawback of PDL was a high unspecific binding of the ligand to the positively charged surface. Such a problem is likely dependent on the properties of the ligand and the use of ligands with higher isoelectric points may solve this issue. Confocal images suggested that fibronectin coatings might boost bacteria attachment, but would need to be optimized to ensure a more homogeneous bacterial immobilization. Among those tested methods, only the use of cell culture dishes was more promising, but resulted in lower signals for both *E. coli* strains CJ236 and BL21 compared to the antibody-based approach (BL21 47 ± 12%, CJ236 64 ± 6%, *n* = 2). The low signals for BL21 was explained by a rapid bacteria detachment from the dish, with only 52% (± 21%, *n* = 4) of FITC-labeled bacteria remaining after 10 h, in contrast to 75% (± 6%, *n* = 3) for CJ236. The CJ236 measurements were repeatable, but the kinetics of the FITC-Ab99–*E. coli* interaction deviated significantly from the kinetics of the antibody-attached bacteria (Supplemental Fig. S2A). This was most evidently observed during incubation, in the association phase, in which a more pronounced curvature was detected. The difference may possibly be due to the bacteria being affected by the attachment processes, changing the antigen levels on their surfaces to some extent. The low capacity and rapid detachment of BL21 made it impossible to obtain accurate binding curves using FITC-Ab99. In conclusion, the three tested alternative methods were inferior to the antibody-based immobilization, but might be regarded as alternatives that could be optimized in case surface antigens for immobilization are not available.

## Discussion

Time-resolved binding curves provide crucial insights into the kinetics and potentially also the binding mechanisms for a receptor-ligand or receptor-drug interaction. We have previously demonstrated the value of RT-CBA on living human cells in cancer research (Björkelund et al. 2011). In this study, we explored the possibilities of expanding the use of RT-CBA to prokaryotic cells. The goal was to find a procedure that ensured that most of the bacteria remained firmly attached for at least 15 h without affecting their viability. Such a method should also be relatively inexpensive, straightforward, and general.

Passive adsorption of antibodies is a well-established strategy to capture antigens for any bio-analytical application with ELISA as a prominent example. Here, we demonstrated that this approach can also be used to immobilize bacteria on the surface of Petri dishes, if suitable surface-accessible antigens are known. The method was successful for both Gram-negative and Gram-positive bacteria. Since the interaction between bacteria and adsorbed antibody is most likely multivalent, it is expected that the bacteria attachment is significantly more stable than the interaction between bacteria and the FITC-Ab99 in solution. If that is the case, then most of the observed signal decrease during the dissociation phase (~ 25% during 10 h) is caused by the dissociation of FITC-labeled antibody, but not from bacteria detachment. However, measurements of FITC-labeled *E. coli* suggested that 4–33% of the bacteria detached from the dish after 10 h of rotation in LigandTracer. It is thus possible that the FITC labeling, albeit superior to the SYTO-9 staining, is not optimal for attachment characterization either, since the fluorophore could potentially alter the surface properties of the studied bacteria.

The detachment of bacteria will have an impact on the interaction characterization if the dissociation rate is in a similar timescale as the detachment rate. For example, a potential bacteria drop of 25% over 10 h (which is likely an overestimation) would affect the ability to determine dissociation rate constants lower than approximately 10^−5^ s^−1^. LigandTracer instruments have accurately characterized *k*_d_ values of 2 × 10^−6^ s^−1^ in the past (Bondza et al. 2017), but 10^−5^ s^−1^ is similar to what is considered the practical limitations of SPR systems (Yang et al. 2016). According to a survey of the kinetics and affinities of 33 drugs and drug candidates, most drug interactions have dissociation rate constants of 10^−5^ or higher (Dahl and Akerud 2013) meaning that the practical effect of the dissociation rate limitation induced by bacterial detachment is moderate in the field of drug discovery.

Attachment of bacteria through adsorbed antibody requires the availability of an antibody with stable interaction with the cells. The polyclonal Ab99 used a large number of intact and lysed *E. coli* strains as immunogens, making it “reactive with all O and K antigenic serotypes of *E. coli*,” according to the manufacturer. For other Gram-positive or Gram-negative bacteria, specific antibodies have to be found for each particular type. However, if bacterial surface-display systems are available, the use of an established anti-His antibody to capture surface-displayed poly-His-tag is a fit option. Thus, the described antibody-based approach could allow any surface-display system to be effectively immobilized on the Petri dish.

The use of cell culture dishes was both faster and cheaper (1 USD/dish) compared to antibody adsorption (3–9 USD/dish with 1–3 target areas), but resulted in lower signals, in particular with the *E. coli* strain BL21. Measurements with FITC-labeled bacteria also indicated that BL21 quickly detached from the cell culture dishes. One of the major differences between the two *E. coli* strains used in this study is that BL21 lacks flagella, which may then be crucial for the attachment to cell culture dishes. This suggests that the method relying on cell culture dishes is less general, but may still be a convenient alternative for strains that do attach to the dishes and when the signal height is not a limiting factor.

The ability to control bacterial attachment has clear advantages in RT-CBA and other analytical procedures. Even though several attachment methods have been described in the past, the requirements for continuous monitoring of bacteria over many hours constitute a novel set of boundary conditions. The need for long reliable measurements is underrated, but in view of that pharmaceuticals often stay in circulation hours or even days, it is of the essence to match the analytical preclinical procedures to the time frame of biological action in the target organism. Hence, the results in this report are enabling for precise analyses in biologically relevant timescales in microbiology, similar to what is already applied in preclinical oncology. As a consequence, most of all other assay possibilities that are already developed for the LigandTracer platform become available for the microbiological field, such as time-resolved “dimerization” assays (Bondza et al., 2017), Motulsky-Mohan-type competition assays (Motulsky and Mahan 1984) and time-resolved viability assays (Vennström et al. 2008).

In conclusion, we believe that the attachment of bacteria through adsorbed antibody is a suitable approach when there is a need for immobilizing prokaryotic cells. This method enables measurements in RT-CBA such as LigandTracer, which may provide much more information about the kinetics of interactions with bacteria. The antibody-based attachment procedure worked efficiently for all tested bacterial species and strains, both Gram-positive and Gram-negative, suggesting that the method is general if a relevant antibody is available. Attachment through antibodies is not restricted to RT-CBA measurements and this strategy may also be applied to other microbiological methods requiring immobilized bacteria, such as in microscope studies.

## Electronic supplementary material

ESM 1 (PDF 354 kb)
