# Supplementary material for: Detecting ligand interactions in real time on living bacterial cells
Source: Appl Microbiol Biotechnol. 2018 Mar 17;102(9):4193–201. doi: 10.1007/s00253-018-8919-3 (PMC5895670; doi:10.1007/s00253-018-8919-3)
Supplement: Supplementary file 1 — (PDF 354 kb) [file 253_2018_8919_MOESM1_ESM.pdf]

Applied Biotechnology and Microbiology

Detecting ligand interactions in real-time on living bacterial cells

Crispim Encarnação, João[a,b], Schulte, Tim[c], Achour, Adnane[c], Björkelund, Hanna[a], Andersson, Karl[a,b]

Affiliation:

[a] Ridgeview Instruments AB, Vänge, Sweden

[b] Department of Immunology, Pathology and Genetics, Uppsala University, Sweden

[c] Science for Life Laboratory, Department of Medicine Solna, Karolinska Institute, and Department of Infectious Diseases, Karolinska University Hospital, Solna, Sweden

Corresponding Author:

João Encarnação, Ridgeview Instruments AB, Vänge, Sweden

E-mail: joao@ridgeview.eu

Phone: +46 73-073 54 83 (CET)

Supplementary material

Results

Optimization of passively adsorbed antibodies

Polyclonal rabbit anti-*E. coli* antibody (ab31499, denoted Ab99) was adsorbed in circular ~3 cm^2 areas of a polystyrene dish and a cell culture dish at different concentrations (3 µg/mL or 6 µg/mL). The binding of 4.4 nM of an Alexa 488 labeled goat anti-rabbit IgG antibody to the coated areas was studied in real-time with LigandTracer Green, to confirm and estimate the amount of adsorbed Ab99, as well as to study the stability of the adsorption (Supplemental Fig. S1A). As expected, the hydrophobic polystyrene dish was found to be more suitable for adsorption and produced more than thirteen times higher binding signal than the cell culture dishes after 2 h of incubation with the secondary antibody. Approximately 30 % more anti-rabbit IgG bound to the 6 µg/mL spot than to the 3 µg/mL spot. The non-linear relationship between antibody concentration and amount of adsorbed antibody may be the result of surface saturation. After a few hours of incubation with the fluorescent anti-rabbit IgG antibody, the solution was replaced with fresh buffer in the polystyrene dish. The signal dropped 9-10 % during the first 10 h of the dissociation measurement, due to a combination of the anti-rabbit IgG dissociating from Ab99 and a possible detachment of adsorbed Ab99 from the dish. In other words, no more than 10 % of Ab99 can be expected to detach during 10 h of rotation in LigandTracer.

Once the adsorption of the Ab99 had been confirmed, the attachment of bacteria could be tested. Bacteria from the *E. coli* strain BL21 were incubated with adsorbed Ab99 on three spots within the same dish, using different antibody and bacteria concentrations (Spot A: 3 µg/mL Ab99 at OD 1.0, Spot B: 6 µg/mL Ab99 at OD 0.35 and Spot C: 6 µg/mL Ab99 at OD 1.0). The binding of 10 and 30 nM FITC labeled Ab99 to the attached bacteria were monitored in parallel with LigandTracer Green (Supplemental Fig. S1B). It was possible to detect a binding of FITC-Ab99 to the spots, indicating the presence of *E. coli* bacteria. The 10 nM signals were too low for a proper comparison, but the association curves produced with 30 nM FITC labeled Ab99 indicated that there may be 30-35 % more attached *E. coli* at Spot C (Supplemental Fig. S1B, black). This combination of antibody concentration and bacteria density was selected for the rest of the study since it was speculated that the signal height may be a limiting factor.

Suitable adsorption times were investigated by adsorbing Ab99 for 3 h, over-night and 4 days and then comparing the signal of FITC-Ab99 to Ab99-attached bacteria. It was found that over-night and 4 day adsorptions may sometimes reduce the biological activity of Ab99 (data not shown) and it was decided to use an adsorption time of 3 h.

Alternative immobilization methods

Fibronectin coatings to attach bacteria has been described in the literature and was tested as it may be of interest for other bacteria than *E. coli* as well. Different coating times were compared (over-night, 1 h and 3 h) and it was found that 3 h produced the best result in terms of signal height (data not shown). The interaction data of the FITC labeled

Ab99 binding to attached bacteria were repeatable and produced similar kinetics than the adsorbed antibody approach, for both tested strains (Supplemental Fig. S2B). However, it was found that the signal levels were similar than when simply attaching the bacteria to uncoated polystyrene dish, hence the fibronectin did not improve the overall attachment. With confocal imaging it was found that fibronectin can boost bacteria attachment, but only in limited clusters (Supplemental Fig. S2C).

The signals from uncoated polystyrene dishes were less than half of the signals from the adsorbed antibody approach (CJ236: 43 ± 3 %, BL21: 34 ± 2 %, n = 2, measured at t = 15 h). Approximately 3/4 of the signal from FITC-labeled bacteria remained after 10 h of measurement (CJ236: 72 ± 2 %, BL21: 85 ± 1 %, n = 2).

Another approach for bacteria attachment mentioned in the literature is the use of polylysine. The amount of bound FITC-Ab99 was high for all tested PDL coatings, even higher than to the bacteria attached with adsorbed Ab99. Unfortunately, it was found that the signal height was comparable to the signal from bacteria free PDL coated areas, indicating unspecific binding of FITC-Ab99 to PDL. Confocal images did however show a large amount of bacteria in the PDL coated areas (Supplemental Fig. 2C) so this approach may be suitable for ligands that do not stick to PDL.

Supplemental figures

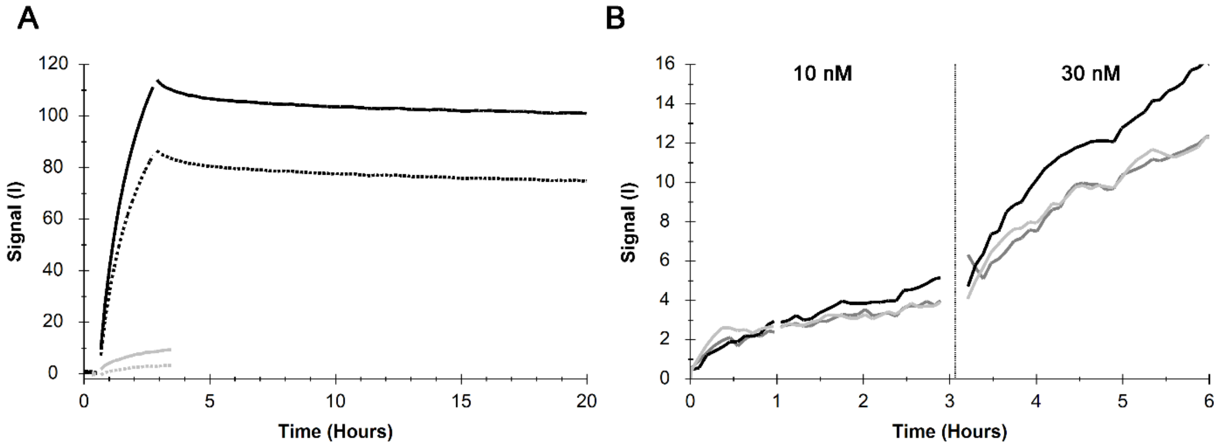

Supplemental Fig. S1. A) Binding of Alexa 488 labeled anti-rabbit Ig G antibody to rabbit anti-*E. coli* antibody (Ab99), adsorbed at concentrations of 6 µg/mL (solid lines) or 3 µg/mL (dotted lines) in polystyrene dishes (black) and cell culture dishes (grey). B) 10 and 30 nM FITC labeled anti-*E. coli* antibody binding to the attached *E. coli* strain BL21, using different antibody and bacteria concentrations: 6 µg/mL Ab99 at OD 1.0 (black), 6 µg/mL Ab99 at OD 0.35 (dark grey) and 3 µg/mL Ab99 at OD 1.0 (light grey).

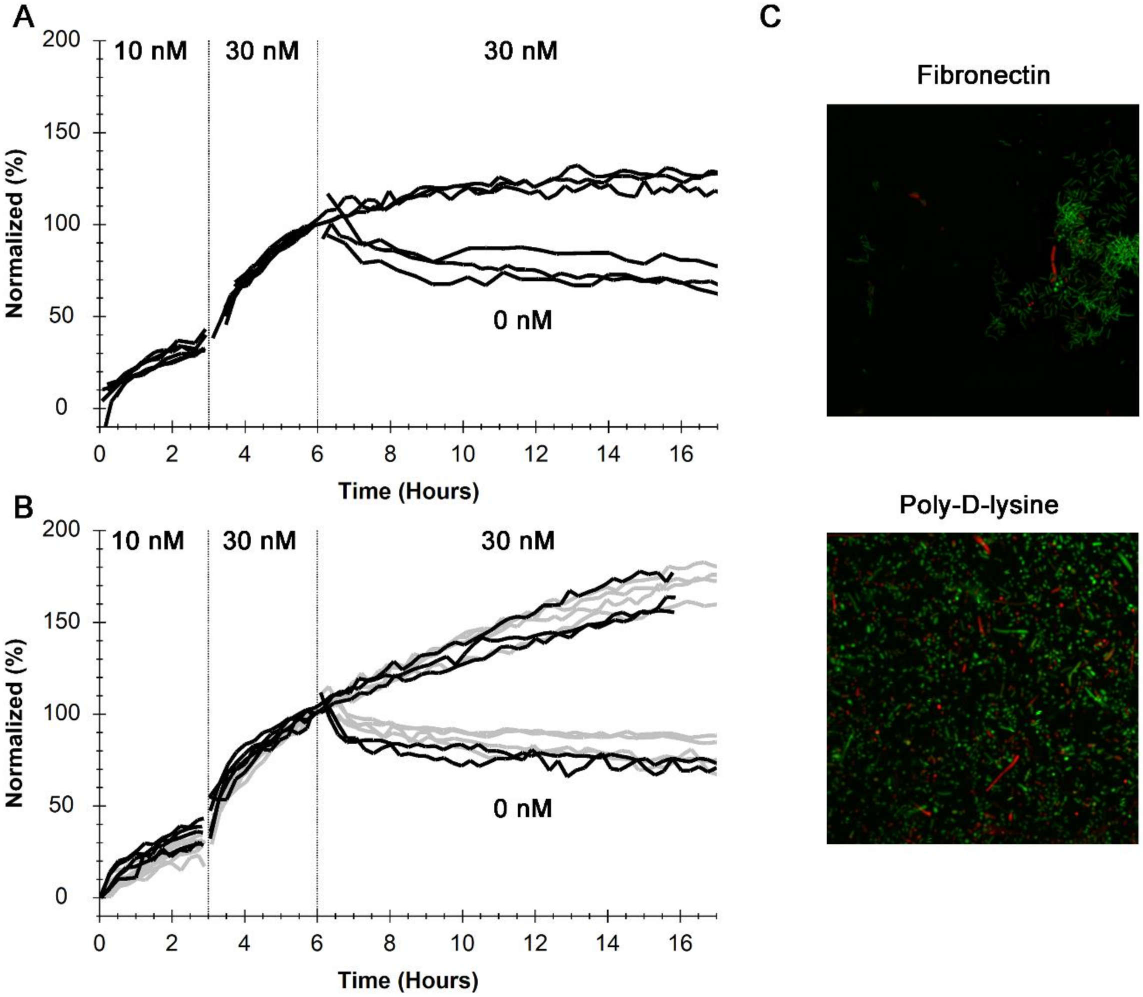

Supplemental Fig. S2. A, B) Normalized association and dissociation curves of FITC labeled Ab99 to *E. coli* strains CJ236 (black) and BL21 (grey) attached to cell culture dishes (A – only CJ236) or to fibronectin coated polystyrene dishes (B). n = 2-4 for each combination of strain, attachment approach and incubation variant (= with/without dissociation phase). C) Confocal images of living (green) and dead (red) cells after 16 h on a rocker, when attached to fibronectin or poly-D-lysine.
